# Supplementary material for: Comparison of gene clustering criteria reveals intrinsic uncertainty in pangenome analyses
Source: Genome Biol. 2023 Oct 30;24:250. doi: 10.1186/s13059-023-03089-3 (PMC10614367; doi:10.1186/s13059-023-03089-3)
Supplement: Supplementary file 5 — Additional file 5. Review history. [file 13059_2023_3089_MOESM5_ESM.docx]

**1^st^ round**

**Reviewer 1**

The authors describe a comparative analysis of several methodological approaches for building pangenomes. The manuscript is very well-written. The results of these types of comparisons can be crucial. Additionally, the authors were thorough in their comparisons and described them well, including attractive and easy-to-understand figures. However, the analyses are incomplete for several reasons (described below).

Moreover, to publish such a manuscript in a journal of the caliber of Genome Biology, I would expect the authors to prepare a manuscript that is so definitive that it is the authority on approaches. As is, the authors do a fantastic job of introducing the challenge, but there are no data related to new biological discoveries, nor do the authors describe a novel method. Yet, as described below, the broadened analyses could be important contributions to pangenome research.

Major Revisions

1. A comparative analysis of methods in relatively new and evolving fields can help justify future method choices. Delineating the relative strengths and weaknesses can help researchers understand what they may confidently conclude from the results or not. The value, then, is in clearly and explicitly articulating these differences. The authors clearly describe their work. However, some important items are missing from both the comparisons and what is included in the manuscript if the authors wish for this manuscript to be the de facto authority in pangenome analyses:

a. The authors describe the test dataset as being full of relatively complete genomes. This represents a best-case scenario for people doing pangenome research and the most straightforward test case. I believe the inclusion of these data is essential. However, including more realistic datasets is critical to thoroughly testing the methods. Some examples include incomplete or poorly assembled genomes, mixing incomplete genomes with complete genomes, etc. The authors can likely think of additional ways to make their comparisons/testing more broadly applicable. To be helpful, researchers need to know how the described methods will work on their dataset, which is likely to be less clean that what has been used for testing here—otherwise are forced to extrapolate results in a way that may or may not be helpful.

b. In a manuscript such as this, I'd expect a table clearly articulating each approach's strengths and weaknesses (even if such detail is already present in the text). I'd also hope the authors make specific recommendations about when to use which approach.

c. The authors mention that the choice of genome assembly matters. How about including genomes that were assembled with different approaches too?

d. I understand the importance of maintaining reasonable computational costs and the subsequent subsampling, but if you included all the genomes (even more than 100 per species), would it change outputs? (One can imagine an analysis considering the pangenomes based on 10, versus 20, versus 30, etc. complete genomes to see how widely sampled subspecies need to be before capturing everything, or almost everything—I'm not asking for this as it's tangential, but it would be interesting.)

2. The authors mention that the results are relatively small but reproducible. From published studies, can you identify examples from the primary literature where you reanalyzed data using your recommended approaches and the results meaningfully changed?

3. The authors correctly point out the importance of carefully selecting clustering criteria in the Discussion, Practical Guidelines section. Based on the analyses, what do the authors recommend?

4. The authors describe the importance of developing multi-method approaches. Developing such an approach/database wasn't the authors' purpose for this study (although, to be frank, I think the contribution of their work would be significantly higher if they had); what if they combined different approaches in different ways?

Minor Revisions

1. Listing the exact parameters used for each software in a single table in the supplement could be helpful for those attempting to use the same approaches instead of just in the text.

2. I think it's entirely appropriate not to have included every possible algorithm but rather choosing representative implementations of each different methodological approach. However, a supplemental table listing widely used tools would be helpful.

**Reviewer 2**

In this manuscript, the authors test the performance of different categories of pangenome software and determine that they produce different results, especially in terms of genome fluidity. The study is interesting and much needed to better understand the performance of pangenome tools available. I do not have major issues with the study but I would like the authors to clarify the concept of fluidity and its relation to accessory genomes. From my understanding, genome fluidity is a measure that was created to provide an alternative to pangenomes when incomplete genomes are available because fluidity is not as sensitive to partial sampling. The higher the level of fluidity, the less similar two genomes are. However, this is a measure that is similar to the accessory genes measure from pangenomes. How do the authors explain the different consistency in performance of accessory genes and fluidity across the different methods? I suspect that the difference rests exactly in the nature of the fluidity, that is calculated from subsamples of the data instead of the full data. In such a scenario, the variation observed by the authors would be spurious, created by the usage of incomplete datasets that violate the core principle of pangenomes, which is the representation of all genes in the genomes analyzed. I would encourage the authors to clarify what effect the random subsampling they applied might have had on the variance in fluidity observed and what kind of implications this variance might have on downstream analyses.

**Authors’ response**

*Reviewer #1: The authors describe a comparative analysis of several methodological approaches for  building pangenomes. The manuscript is very well-written. The results of these types of comparisons  can be crucial. Additionally, the authors were thorough in their comparisons and described them  well, including attractive and easy-to-understand figures. However, the analyses are incomplete for  several reasons (described below).*

*Moreover, to publish such a manuscript in a journal of the caliber of Genome Biology, I would expect  the authors to prepare a manuscript that is so definitive that it is the authority on approaches. As is,  the authors do a fantastic job of introducing the challenge, but there are no data related to new  biological discoveries, nor do the authors describe a novel method. Yet, as described below, the  broadened analyses could be important contributions to pangenome research.*

We thank the reviewer for the positive comments and constructive criticism. Following the  reviewer’s suggestions, we have added a whole new section, four figures (one in the main text and  three in the supplementary material) and three tables that substantially expand the scope of the  manuscript. As a result, we expect that the revised version provides the deep insight and high level  of applicability required for publication in Genome Biology.

*Major Revisions*

*1. A comparative analysis of methods in relatively new and evolving fields can help justify future  method choices. Delineating the relative strengths and weaknesses can help researchers understand  what they may confidently conclude from the results or not. The value, then, is in clearly and explicitly  articulating these differences. The authors clearly describe their work. However, some important  items are missing from both the comparisons and what is included in the manuscript if the authors  wish for this manuscript to be the de facto authority in pangenome analyses:*

*a. The authors describe the test dataset as being full of relatively complete genomes. This  represents a best-case scenario for people doing pangenome research and the most straightforward  test case. I believe the inclusion of these data is essential. However, including more realistic datasets  is critical to thoroughly testing the methods. Some examples include incomplete or poorly assembled  genomes, mixing incomplete genomes with complete genomes, etc. The authors can likely think of  additional ways to make their comparisons/testing more broadly applicable. To be helpful,  researchers need to know how the described methods will work on their dataset, which is likely to be  less clean that what has been used for testing here—otherwise are forced to extrapolate results in a  way that may or may not be helpful*.

We completely agree that it is important to test the performance of gene clustering methods in more  realistic scenarios, because that will help researchers make beter choices depending on the quality  of their datasets. For that reason, we have extended the study to pangenomes built from incomplete  genomes. In the revised version, we investigate the effect of replacing 5%, 20%, 50%, and 100% of  the original high-quality genomes by medium- or low-quality genomes assembled from  metagenomes (MAG). To keep the computational cost manageable, we focused on 4 representative  species (*Escherichia coli*, *Bacteroides uniformis*, *Staphylococcus epidermidis*, and *Cutibacterium  acnes*) that belong to 4 major phyla and cover a broad range of pangenome sizes and diversities. Overall, we built 96 new pangenomes (4 species x 4 proportions of MAG x 2 quality levels x 3 random

subsamples of each condition), almost duplicating the total number of pangenomes in the study. By  analyzing these pangenomes, we found that estimates of pangenome size and gene content diversity  are more or less robust to the inclusion of incomplete genomes depending on the criterion used for  gene clustering. In almost all cases, reference-database mapping produces the most consistent  results, whereas orthology produces the least consistent results. To provide quantitative guidelines  for method selection, we determined the maximum fraction of incomplete genomes that can be  included in the pangenome before its inferred properties become substantially biased. We also  explored the usefulness of Bayesian approaches (like those implemented by mOTUpan) to improve  the estimation of gene content diversity from medium- and low-quality pangenomes. The results of  these analyses are presented in a new subsection (“Estimation of pangenome properties from  medium- and low-quality genome assemblies”) and 4 new figures (6, S5, S6, and S7). Moreover, we  have included specific advice to deal with incomplete genomes in the Discussion section and in a new table (Table 2).

*b. In a manuscript such as this, I'd expect a table clearly articulating each approach's strengths  and weaknesses (even if such detail is already present in the text). I'd also hope the authors make  specific recommendations about when to use which approach*.

Following the reviewer’s suggestion, we added a new table (Table 2) that summarizes the strengths and limitations of each approach. The table also includes specific recommendations about when to  use each approach, according to the intended research purpose and the quality of the genome assemblies. We hope that the table will serve its purpose as a handy summary of the practical  guidelines presented with more detail in the text.

*c. The authors mention that the choice of genome assembly matters. How about including  genomes that were assembled with different approaches too?*

Among the many, often nuanced, differences associated with the choice of genome assemblers, varying levels of completeness and contamination are those that most directly affect the properties  of the resulting pangenomes. For that reason, rather than conducting an explicit comparison of  assembly tools, we focused on studying the joint effects of genome completeness and  contamination, as embodied by the overall assembly quality, in a particularly challenging scenario: genomes assembled from metagenomes. We believe that this approach is especially relevant, given  that MAG have become a major source of genomic data for non-model bacteria. Moreover, thanks  to the broad range of assembly qualities covered by public collections of MAG, we could compare  pangenomes built from medium- and low-quality genomes, without having to reassemble the whole  dataset. The results of these analyses are included in the new subsection “Estimation of pangenome  properties from medium- and low-quality genome assemblies”.

*d. I understand the importance of maintaining reasonable computational costs and the  subsequent subsampling, but if you included all the genomes (even more than 100 per species),  would it change outputs? (One can imagine an analysis considering the pangenomes based on 10,  versus 20, versus 30, etc. complete genomes to see how widely sampled subspecies need to be before  capturing everything, or almost everything—I'm not asking for this as it's tangential, but it would be  interesting.)*

We agree that connecting our comparison of gene clustering methods to a systematic study of  sampling effects would be interesting. However, the task is challenging enough (both conceptually  and practically) to merit a separate study. As an example of such conceptual complication, consider  the following apparently straightforward task: to compare gene clustering methods by determining  the smallest number of genomes required to produce sufficiently robust estimates of pangenome  properties. One could naively argue that the best method is the one that allows estimating the  property of interest from the smallest possible number of genomes. However, such faster  convergence could also mean that the method is unable to distinguish among paralogs or remote  homologs, in which case it may be beter to use a different tool. Therefore, a simple ranking of gene  clustering criteria based on their sampling efficiency would not be as informative as it could appear.

Those caveats aside, we took especial care to ensure that our results are robust with respect to  differences in the number of genomes available for each species. Thus, for each pangenome feature,  we initially calculated its raw value (using all available genomes up to 100), the average over 100  random subsamples of 14 genomes, and the value for the 15 most divergent genomes. Moreover, in  the case of pangenome size, we applied two alternative corrections aimed at reducing the  dependency on the number of genomes. We found that all these metrics were highly correlated  (R>0.95), concluding that cross-method variability of pangenome estimates remains similar  regardless of sample size correction (or the lack of it).

To confirm the robustness of our results in a more explicit manner, we investigated if cross-method  inconsistencies (Figure 2c-d) depend on the number of genomes used to build each pangenome. We did that by calculating the correlation between the residuals of the cross-method orthogonal  regressions (for example, those in Fig 1d, where each data point corresponds to one species) and  the number of genomes associated with each species. We did not find any significant correlation.  That indicates that cross-method variation is distributed across species in a way that does not  depend on the number of available genomes (at least for the range of 15-100 genomes explored in  this study). Therefore, it is unlikely that increasing or decreasing the number of genomes per species  substantially modifies the observed values of cross-method variability. This information in now  included in the second-to-last paragraph of the subsection “Method-dependent variation and  intrinsic uncertainty in pangenome size and diversity”.

2. *The authors mention that the results are relatively small but reproducible. From published  studies, can you identify examples from the primary literature where you reanalyzed data using your  recommended approaches and the results meaningfully changed?*

We actually found some differences between our results and those of previous studies. The most  notable one concerns the (lack of) correlation between the gene content diversity (measured, for  example, by the genome fluidity) and the nucleotide diversity of the core genome. A previous  publication by Maistrenko et al. (Ref. 6 in the manuscript) reported that both measures were closely  related and could be jointly interpreted in terms of pangenome diversity. Our study did not find such  a close relationship. Instead, as shown in Fig 2b, core nucleotide diversity and gene content diversity  constitute two clearly differentiated properties, regardless of the criterion used to define the OGC. The lack of association between these two properties makes perfect sense because the ratio  between nucleotide substitution rate and gene turnover rate is extremely variable across species

(see, for example, Ref. 43 in the manuscript).

Although we tried to track the causes for the disparity between our results and those in Ref. 6, the  lack of technical details in Ref. 6 made it impossible to sort out a single cause. Three possible sources  of variability between both studies are: (i) the selection criteria for high-quality genomes, that were  more stringent in our study (requiring completeness >99% and contamination <1%, instead of the  standard 90% and 5% thresholds); (ii) the number of genome pairs used to calculate fluidity, that we  increased from 10 (the default value in micropan) to 500 in order to eliminate sampling noise; and  (iii) the method used to calculate the core nucleotide diversity, that in our case was based on all against-all Needleman-Wunsch pairwise alignments. Although Ref. 6 does not specify how the  nucleotide identity was calculated, the default for Roary is to generate multiple alignments with

PRANK, which can produce biased estimates if internal branches in the strain tree are short. Based  on our observation that genome incompleteness leads to overestimation of genome fluidity, an  artifactual association between core nucleotide diversity and genome fluidity could be expected if  the pangenomes that include more divergent strains (in terms of nucleotide substitutions) also  include more incomplete genomes.

After giving it much thought and considering the length of the manuscript, we decided to present  our results in a straightforward manner, as we do in Fig. 2b and the main text. We believe that openly  engaging in a controversy with the authors of Ref. 6 will divert from the main message of the  manuscript without adding much value. Nevertheless, if the reviewer considers that this is an  important point that should be explicitly discussed in the manuscript, we are open to reconsidering our decision.

3. *The authors correctly point out the importance of carefully selecting clustering criteria in the  Discussion, Practical Guidelines section. Based on the analyses, what do the authors recommend?*

In the light of this comment, we realized that our intended recommendations in the Practical  Guidelines section were not sufficiently explicit. In the revised version, we used a more direct  language to ensure that practical recommendations are clear enough. Moreover, following our  analysis of MAG, we now provide suggestions based not only on the research goal (identifying core  genes, studying genome plasticity…) but also on the quality of the genome assemblies. For greater  clarity, our recommendations are summarized in Table 2.

More generally, through the whole manuscript we have tried to convey a two-fold message. First,  different clustering criteria are appropriate for different purposes (with Table 2 listing the best  choices in each case). Second, in cases in which there is not a clear conceptual basis to choose one criterion over the rest, several methods should be compared to ensure that the conclusions are  robust.

4. *The authors describe the importance of developing multi-method approaches. Developing such  an approach/database wasn't the authors' purpose for this study (although, to be frank, I think the  contribution of their work would be significantly higher if they had); what if they combined different  approaches in different ways?*

A major conclusion of our study is that there is no “one size fits all” gene clustering method for  pangenome analysis. That does not only reflect a practical limitation, but a conceptual one. For  applications that involve tracking vertically transmited genes, the best OGC are those that  effectively discriminate among in-paralogs. (That is the reason why synteny OGC are preferable to

study single-copy core genes.) However, those same OGC are not optimal to study within-species  expansions and contractions of accessory gene families (which requires keeping in-paralogs together  but separated from out-paralogs). This fundamental incompatibility must be taken into account  when combining different approaches.

The solution that we propose is to leverage the hierarchical nature of gene clustering criteria, with  synteny OGC nested within orthology OGC, and the later nested within homology OGC. Such multi- level clustering scheme would facilitate selecting the best set of OGC for each purpose and assessing  the robustness of pangenome properties across gene clustering levels. The multi-method dataset  that we produced in this study, though not strictly nested, constitutes a first step in that direction.

We have rewriten the last paragraph of the manuscript to present our proposal for a multi-level,  nested clustering scheme. Given that some state-of-the-art tools for pangenome analysis, such as  PEPPAN, already combine modules for orthology- and synteny-based gene clustering, we believe  that other research groups dedicated to software development would be interested in using our  proposal to create the next generation of tools.

*Minor Revisions*

1. *Listing the exact parameters used for each software in a single table in the supplement could be  helpful for those attempting to use the same approaches instead of just in the text.*

As suggested, we have listed the exact parameters used for each software in Supplementary Table  2.

*2. I think it's entirely appropriate not to have included every possible algorithm but rather choosing  representative implementations of each different methodological approach. However, a  supplemental table listing widely used tools would be helpful.*

The new Supplementary Table 1 includes a formal classification and brief descriptions of some  popular software for gene clustering and pangenome analysis. We acknowledge that, given the large  number of tools and databases developed for that purpose, our selection is necessarily subjective  and arbitrary. For example, we have chosen to omit orthology databases that do not provide a  standalone software for gene clustering. We hope that the list is representative enough of classical  and state-of-the-art methods. However, we will gladly extend the list if the reviewer considers that  some important software is missing.

Reviewer #2: *In this manuscript, the authors test the performance of different categories of  pangenome software and determine that they produce different results, especially in terms of  genome fluidity. The study is interesting and much needed to better understand the performance of  pangenome tools available. I do not have major issues with the study but I would like the authors to  clarify the concept of fluidity and its relation to accessory genomes. From my understanding, genome  fluidity is a measure that was created to provide an alternative to pangenomes when incomplete  genomes are available because fluidity is not as sensitive to partial sampling. The higher the level of  fluidity, the less similar two genomes are. However, this is a measure that is similar to the accessory  genes measure from pangenomes. How do the authors explain the different consistency in*

*performance of accessory genes and fluidity across the different methods? I suspect that the  difference rests exactly in the nature of the fluidity, that is calculated from subsamples of the data  instead of the full data. In such a scenario, the variation observed by the authors would be spurious,  created by the usage of incomplete datasets that violate the core principle of pangenomes, which is  the representation of all genes in the genomes analyzed. I would encourage the authors to clarify  what effect the random subsampling they applied might have had on the variance in fluidity observed  and what kind of implications this variance might have on downstream analyses.*

We thank the reviewer for pointing this out, as this is something that we had indeed considered but  not explained in sufficient detail in the manuscript. We will first answer the second part of the  comment, that is, the concern about the possibility that fluidity estimates vary simply because their  calculation involves a random subsampling of genome pairs. In the previous version of the  manuscript, there was a very succinct comment in the Methods section indicating that fluidity was  calculated by setting the option ‘n.sim = 500’, without any additional explanation or  contextualization. This certainly requires further elaboration, that we provide here and in the revised  manuscript. To ensure that variations in fluidity were not simply due to the randomness of the  subsampling algorithm, we conducted a series of preliminary tests to determine the minimum  number of pairs required to achieve convergence in fluidity estimates. We found that subsampling 500 random pairs (instead of only 10 pairs that is the default in micropan) reduced the relative variability across repeated measurements below 0.5%. Therefore, random variability in the  calculation of fluidity cannot explain the >20% unexplained variation across methods. We added this  clarification to the Methods section. However, if the reviewer considers that it merits an explicit  comment in the main Results section, we can move it there.

Regarding the different consistency of accessory genes and fluidity as measures of gene content  diversity, we would like to clarify that the magnitude that correlates (both conceptually and  empirically) with fluidity is the percentage of accessory genes per genome, not the total number of  accessory genes(the later actually correlates with pangenome size). From a conceptual perspective,  the fundamental difference between pangenome features that relate to size and those that relate  to diversity (understood as genome plasticity or gene content diversity, not the nucleotide-level  diversity of core genes) results from the fact that the former are extensive magnitudes and the later  are intensive magnitudes. In other words, diversity magnitudes are explicitly or implicitly normalized  by genome or pangenome size, and that makes them effectively independent of extensive, size related magnitudes. From an empirical perspective, the independence of size- and diversity-related  features has been highlighted, for example, by Maistrenko et al (ISME J, 2020), and it also becomes  evident from Fig 2b of the manuscript. (To avoid misunderstandings regarding the number of  accessory genes and the percentage of accessory genes per genome, we rephrased the section of  the methods that describes the later as a measure of gene content diversity.) In our study, we  included three features related to pangenome diversity: the fluidity, the average proportion of  accessory genes per genome, and the average proportion of singletons per genome. All of them  show similar levels of inconsistency across methods. For example, the unexplained variance when  comparing synteny and orthology is 21% for fluidity and 17% for the proportion of accessory genes  per genome (these values are color-coded in Fig 2c). In Proteobacteria, the unexplained variance is  larger for the fluidity than for the proportion of accessory genes per genome (54% vs 30%), although  the later still has the second largest uncertainty of all pangenome features. We would also like to

point out that calculating the percentage of accessory genes per genome as an average over 100  random subsamples (as we do in the manuscript to eliminate possible dependencies on sample size)  does not artifactually increase variability. Indeed, the fraction of unexplained variance between  synteny and orthology when using the “gross” percentage of accessory genes per genome  (calculated using all available genomes without subsampling) is 21%, which coincides with the  uncertainty of the fluidity and is very similar to the 17% estimated via subsampling. These  observations indicate that high cross-method variability is intrinsic to the estimation of gene content  diversity, regardless of what specific metrics are used to measure it.

**2^nd^ round**

**Reviewer 1**

I am very pleased with the revised version of the manuscript. The authors thoroughly considered each recommendation and (with what likely required much effort) addressed the points I raised. I believe that this is a strong manuscript appropriate for Genome Biology.

**Reviewer 2**

I would like to thank the authors for taking the time to answer the reviewers' comments to carefully. In my opinion, the changes made have significantly improved the manuscript and I do not have any additional comments.
